# The Impact of Intratumoral Bacterial Response to Vaccine Therapy in Cancer Treatment

**DOI:** 10.3390/ijms27219663

**Published:** 2026-10-28

**Authors:** Yu Wang, Zhong-Jie Sun, Hai-Long Qi, Di Zhao, Xiao-Dong Xing, Jian-Dong Jiang, Tian-Le Gao

**Affiliations:** 1 Institute of Medicinal Biotechnology, Chinese Academy of Medical Sciences & Peking Union Medical College, Beijing 100050, China; 2 Newish Biological R&D Center, Wuxi 214122, China; 3 State Key Laboratory of Elemento-Organic Chemistry, College of Chemistry, Nankai University, Tianjin 300071, China; 4 Institute of Materia Medica, Chinese Academy of Medical Sciences & Peking Union Medical College, Beijing 100050, China

**Keywords:** therapeutic vaccines, HPV, intratumoral microbiota, cancer immunotherapy

## Abstract

Therapeutic Human papillomavirus (HPVs) vaccines are designed to enhance the immunogenicity of HPV and promote the elimination of tumor cells effectively. Mounting evidence implies that tumor-resident microbiomes may influence therapeutic outcomes through immune modulation. In this study, we evaluated whether the intratumoral microbiota affects the efficacy of the therapeutic HPV vaccine. We analyzed RNA sequencing data from tumor tissues of C57BL/6N female mice treated with a DNA vaccine incorporating HPV16 E6/E7 antigens fused with the chemokine CCL11. Microbial community data were acquired using the PathSeq bioinformatics pipeline. Alpha diversity was estimated using the Shannon index and the number of observed taxonomic features, while beta diversity was assessed via principal coordinate analysis (PCoA) based on Bray–Curtis distance. Linear discriminant analysis Effect Size (LEfSe) analysis was performed to identify differentially abundant microbial taxa. Immune cell infiltration was characterized using the xCell deconvolution method. The analysis revealed significant divergence in both the alpha- and beta-diversity metrics of intratumoral microbiota, associated with treatment responsiveness. Vaccinated groups exhibited enriched abundances of the phyla Proteobacteria, together with a concomitant reduction in Firmicutes and Actinobacteria, and were associated with a positive response to cancer immunotherapy. Additionally, intratumoral microbiome composition and diversity correlated with the increased infiltration of CD8^+^ T cells, natural killer T (NKT) cells, and myeloid cells. This study identifies intratumoral microbiota remodeling as a key feature of therapeutic HPV vaccine response. The observed coordination between bacterial signatures and immune cell infiltration is associative and raises the hypothesis that intratumoral bacteria may modulate, rather than drive, antitumor immunity. These findings support the development of intratumoral microbiota profiling and highlight the potential for microbiota-targeted strategies to enhance cancer immunotherapy efficacy.

## 1. Introduction

Human papillomavirus (HPVs), a double-stranded DNA virus that infects the human epithelium, is associated with a wide variety of mucosal and skin lesions, whether benign or malignant [1]. Globally, HPV accounts for over 90% of cervical cancer cases—the fourth leading female malignancy—necessitating routine screening for early intervention [2]. Oncogenic HPV16/18 subtypes dominate cervical carcinogenesis, implicated in 70% of high-grade squamous intraepithelial lesions (HSIL) and invasive carcinomas [3,4]. Epithelial differentiation triggers upregulated viral oncogene expression (E6/E7), rendering these antigens prime targets for therapeutic vaccines that induce tumor-specific cytotoxicity [5,6,7]. Clinical trials (NCT01304524, NCT02596243) demonstrate that HPV antigen-targeted vaccines (E6/E7) generate robust CD8^+^ T cell responses, increasing vaccine immunogenicity against HPV, and correlating with tumor regression in phase II/III studies [6,8,9]. Therapeutic HPV vaccines remain under active clinical evaluation, and a better understanding of the modifiers of vaccine response—including the tumor microenvironment—is needed to further improve clinical efficacy. These findings underscore the need for further research to elucidate the possible mechanisms for determining cellular immunity in patients receiving therapeutic vaccines.

Accumulating evidence suggests that the heterogeneity of the individual microbiomes has a significant impact on the phenotype and therapeutic response of the tumor [10,11,12]. It has been demonstrated that the intratumoral administration of Clostridium novyi induces a profound alteration of the tumor immune microenvironment, converting cold tumors with scant T-cell infiltration into hot tumors [13]. In addition, significant tumor inhibition has been observed in patients with advanced solid tumors following a combination therapy of intratumoral injection of attenuated Clostridium novyi-NT spores and Pembrolizumab. The bacterium exerts its antitumor effect by selectively growing in hypoxic tumor niches, thereby inducing tumor necrosis and an inflammatory response [14]. Meanwhile, most research efforts have concentrated on how intestinal microbiota impacts cancer immunotherapy through immunity activation [15,16]. However, the role of the microbiota in the intratumoral environment needs to be further investigated. Deborah and her colleagues analyzed the microbiota comprehensively, sampling 1526 tumors and adjacent normal tissues from seven different cancers. They found that there was a distinct microbial composition for each type of tumor, with those with more abundant Clostridium perfringens being more sensitive to ICI immunotherapy, while those with high concentrations of Gardnerella vaginalis showed relatively little sensitivity to ICI therapy [12]. In the past, significant differences in intratumoral microbiome markers were observed in patients with Short-Term Survival (STS) and Long-Term Survival (LTS), and the LTS patients showed a higher α diversity in the intratumoral microbiome, suggesting a prognostic value [17]. These results suggest that the intratumoral microbiome may influence host cell homeostasis, tumor development, immune cell activation, and therapeutic efficacy [18,19,20]. Therefore, it is essential for there to be further research to clarify its therapeutic significance and underlying mechanisms in cancer treatment.

Our study reveals that the intratumoral microbiota undergoes dynamic changes in response to therapeutic cancer vaccines. We specifically identified microbial variations linked to immunotherapy outcomes. This study aims to find out if intratumoral microbiota influenced the infiltration level of immune cells, especially CD8^+^ T cells.

## 2. Results

### 2.1. Discovery and Identification of Intratumoral Microbiota in Host RNA-seq Data

**Figure 1:**
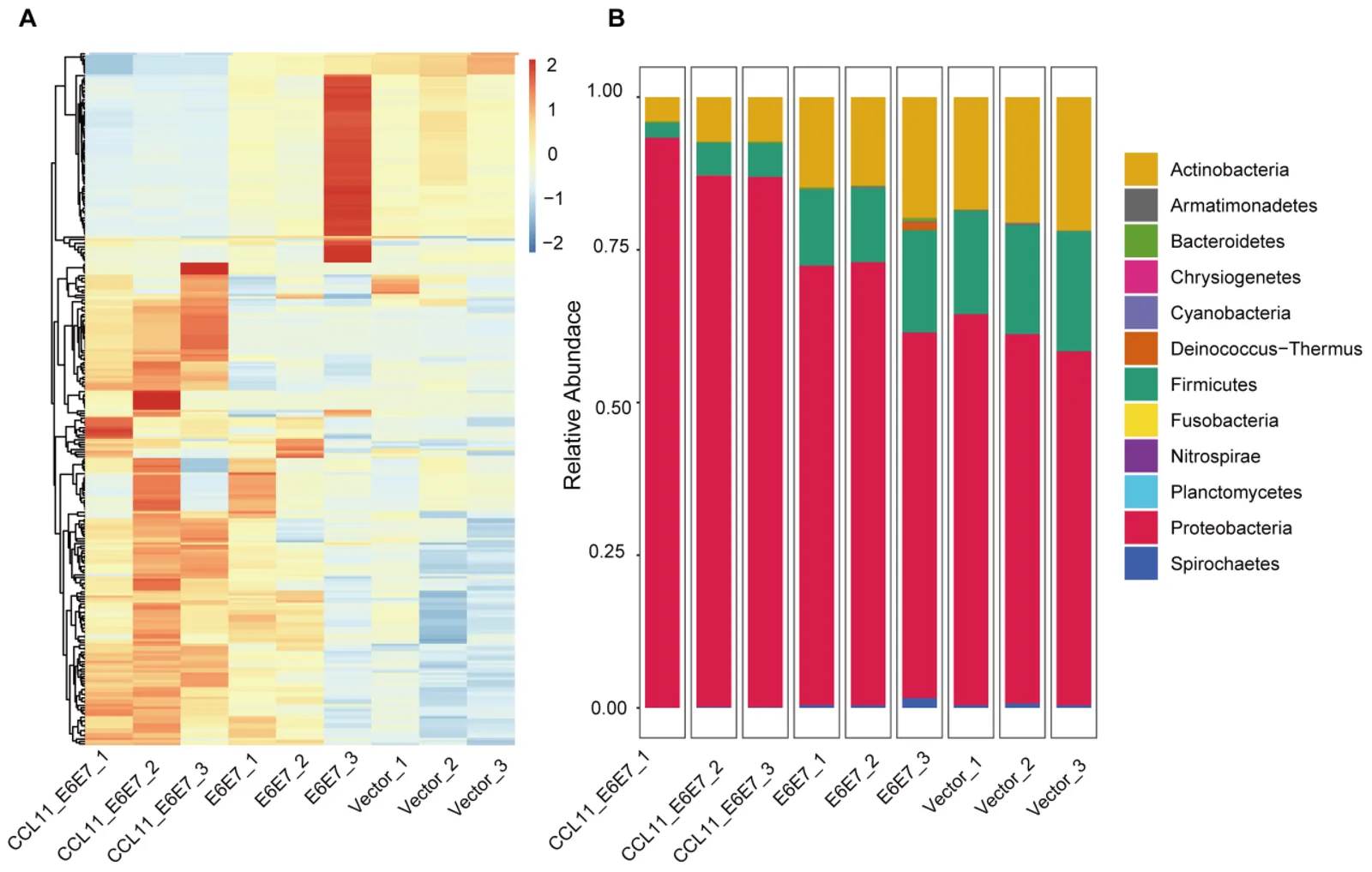
Microbial abundance in vaccinated and control tumor tissues in mice. (**A**) Heatmap visualizing the microbial abundance between the tumor tissues treated with vaccination and control tissues based on hierarchical clustering analysis. (**B**) Composition of microbiota at phylum level in the tumor tissues of vaccinated versus control tissues.

To explore the role of intratumoral microbiota composition in mediating the treatment response to therapeutic vaccination. We used PathSeq, a computational pipeline for the discovery and identification of microbial sequences in host transcriptomic data. Briefly, PathSeq initiates by removing low-quality, low-complexity, host-derived and duplicate reads from the transcriptomic data obtained from a previous study on vaccine immunization [7]. Subsequently, the remaining non-host reads were aligned to a reference database of microbial genomes and classified taxonomically. Ultimately, we generated a report detailing microbial abundance at each taxonomic level. The per-sample numbers of total reads, host-derived reads removed, and retained bacterial reads are detailed in Table S1. The results indicated that significant alterations in overall microbial abundance were detected within the tumor tissues of vaccinated mice relative to non-vaccinated controls (Figure 1A).

### 2.2. Analysis of Intratumoral Microbiota Diversity Following Vaccine Immunization

**Figure 2:**
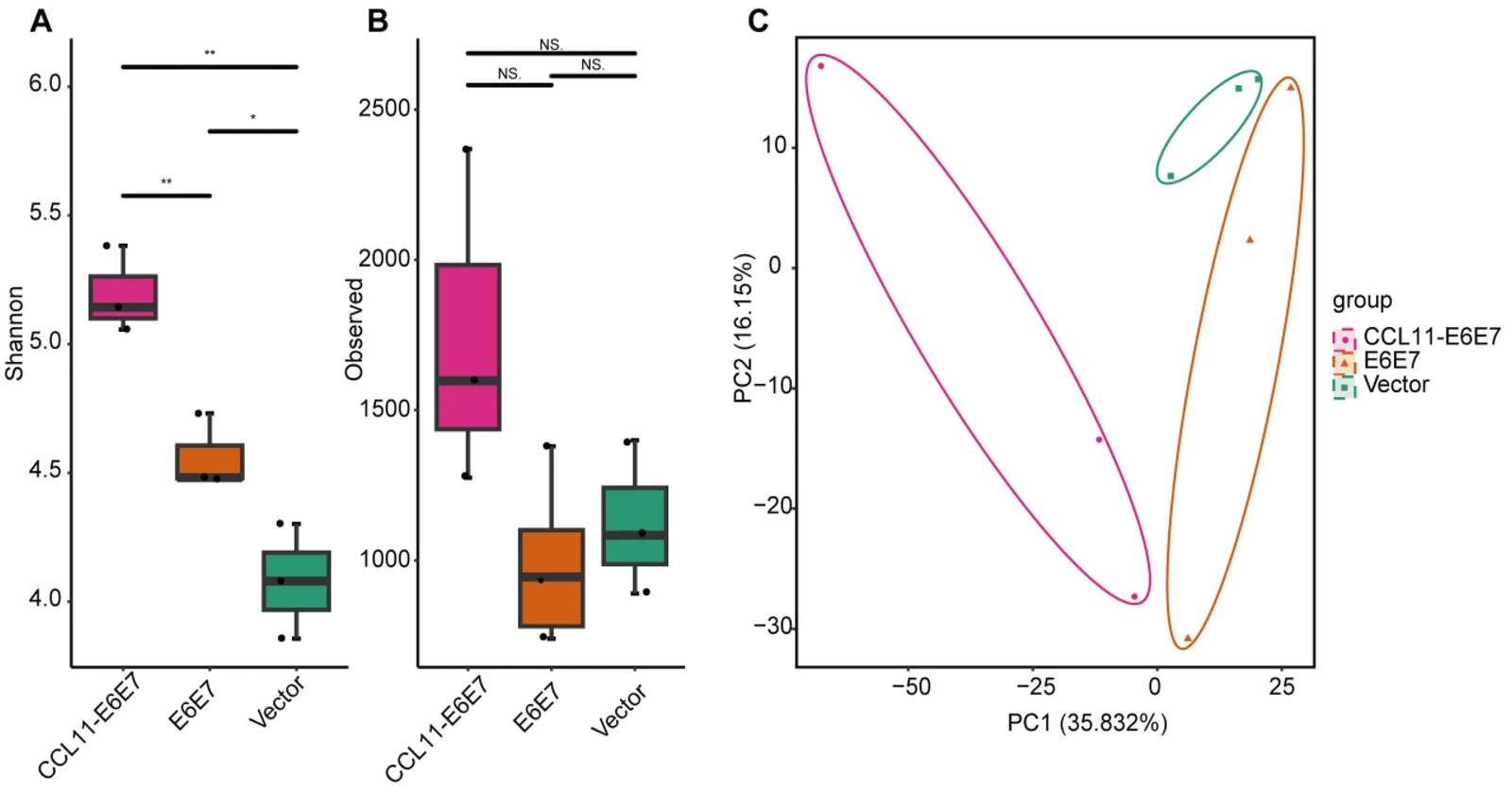
Microbial diversity in vaccinated versus control tumor tissues in mice. (**A**) Microbial alpha diversity, estimated by Shannon and Observed index (**B**), was significantly increased in vaccinated tumor tissues compared with control. (**C**) Beta diversity analysis using PCoA based on Bray–Curtis distance revealed distinct microbiota compositions between groups.( * *p* < 0.05, ** *p* < 0.01).

The classified reads were agglomerated into 12 phyla. We further explored the bacterial diversity between samples that had or had not received vaccine treatment. At the phylum level, *Proteobacteria* predominated in the intratumoral bacterial communities, followed by *Firmicutes* and *Actinobacteria* to a lesser extent. In tumors that received vaccine treatment, particularly with the more effective CCL11_E6E7 vaccine, the abundance of *Proteobacteria* significantly increased, while the abundance of *Firmicutes* and *Actinobacteria* is significantly decreased (Figure 1B). Further analysis revealed diversity differences between the vaccine immunization and control groups. Regarding alpha diversity analysis, tumors treated with the more effective vaccine exhibited the highest number of distinct classified taxonomic features detected and the highest Shannon index value. And significant differences were found between the vaccine treatment groups and the control group in the Shannon index (Figure 2A). Overall, above 1000 classified taxonomic features were detected in the CCL11_E6E7 vaccine treatment groups compared to the control (Figure 2B). Beta diversity, assessed via Bray–Curtis-based PCoA, unveiled profound compositional divergence between the microbiota of vaccinated and control tumors (Figure 2C). The control samples without vaccine immunization clustered closely together, indicating greater homogeneity in their bacterial composition. Samples immunized with different vaccines exhibited significant alterations in the composition of the intratumoral microbiota, and these alterations were associated with the type of vaccine administered. Collectively, the results demonstrated the presence of a distinct intratumoral microbiota signature in samples with vaccine immunization compared to the control.

### 2.3. Identification of Specific Intratumoral Microbial Taxa Changes in Vaccine Immunization

**Figure 3:**
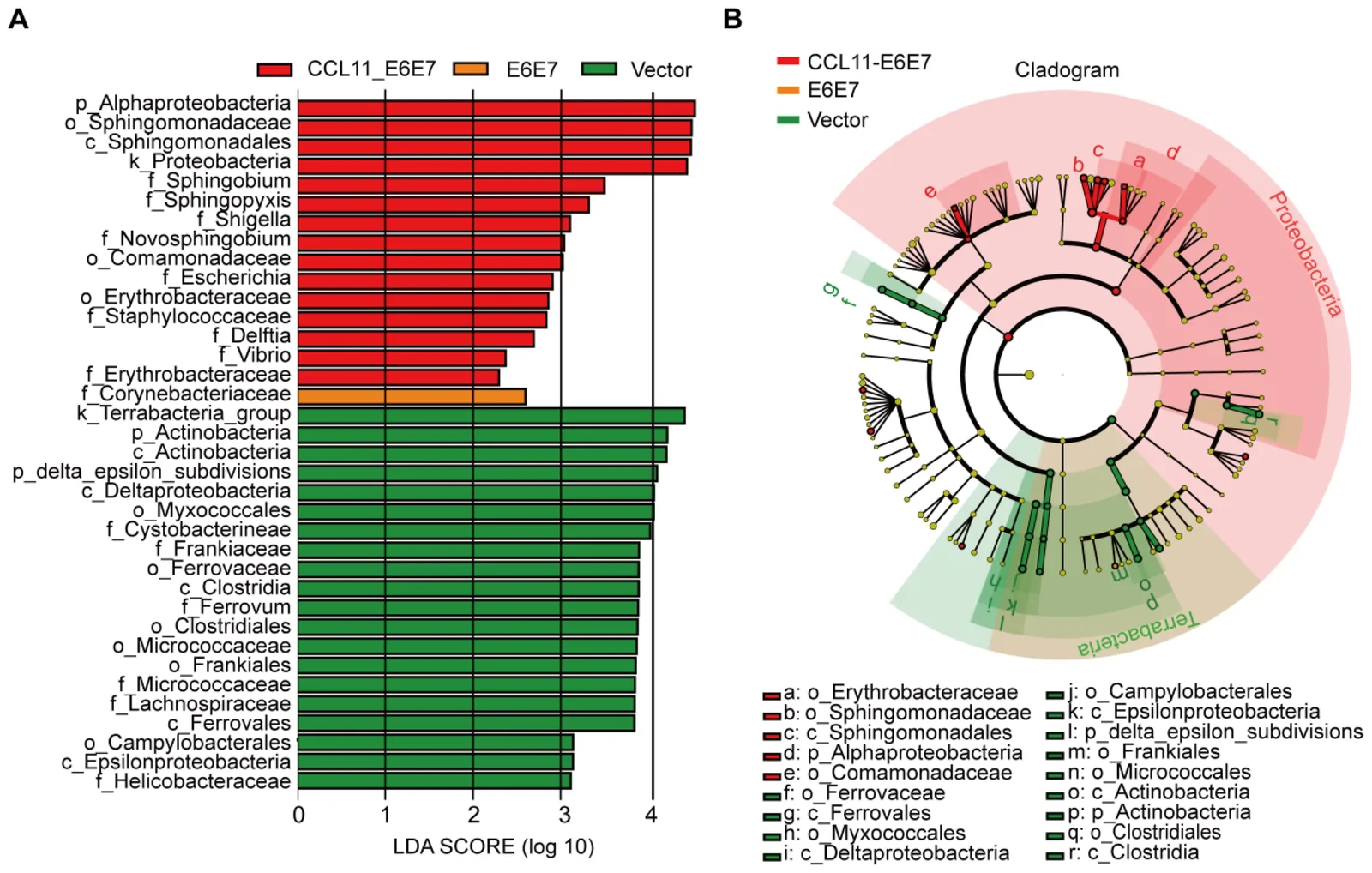
The specific characterization of microbiota between three groups based on LEfSe analysis. (**A**) The histogram of LDA scores showed difference in microbe type and abundance between vaccinated and control groups. (**B**) Cladogram generated by the LEfSe represents the taxonomic hierarchical structure of the identified microbial populations. LEfSe: linear discriminant analysis effect size; LDA: linear discriminant analysis.

We comprehensively profiled the significant differences in intratumoral microbiota between samples with and without vaccine immunization based on relative bacterial abundances. Subsequently, we performed linear discriminant analysis (LDA) with an effect size algorithm and a logarithmic LDA score cut-off >2.0. We identified 15 families that were differentially abundant between the vaccine immunization group and the control group (Figure 3A,B). The vaccine immunization samples exhibited a predominance of *Proteobacteria* taxa, including the families *Sphingobium*, *Sphingopyxis*, *Shigella*, *Novosphingobium*, *Escherichia*, *Delftia*, *Vibrio*, *Erythrobacteraceae,* and *Corynebacteriaceae*, and the orders *Sphingomonadaceae*, *Comamonadaceae,* as well as *Erythrobacteraceae*. In contrast, the unvaccinated control samples were dominated by *Myxococcales*, *Ferrovaceae* and four other orders, as well as the families *Cystobacterineae*, *Frankiaceae*, *Ferrovum*, etc. (Figure 3A). Compared to the control group, the intratumoral microbiomes of the vaccinated group also showed an enrichment in *Proteobacteria* taxa, with the family *Sphingobium* and order *Sphingomonadaceae* identified as significantly enriched (Figure 3B). The most abundant and dominant species within the intracellular environment of a tumor are often crucial in regulating the microenvironment and even the response to immunotherapy [12]. Collectively, these results suggested that the increased presence of *Proteobacteria* might enhance the treatment efficiency of vaccine immunotherapy in treating tumors.

### 2.4. The Tissue-Resident Immune Cell Landscape of the Vaccine Immunization Compared to the Control

**Figure 4:**
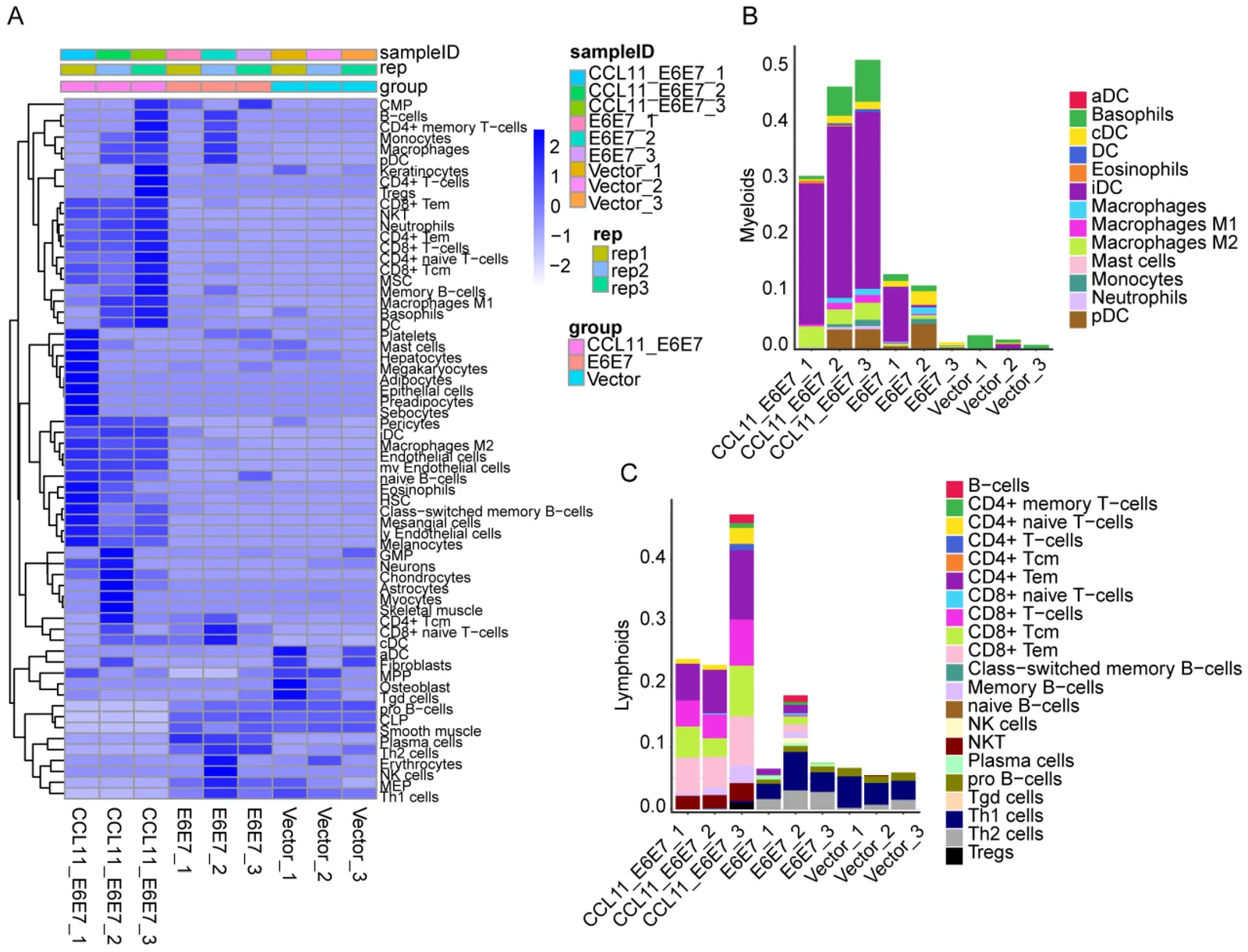
Characterization of tumor-infiltrating immune cells between vaccinated and control groups. (**A**) An overview of adjusted scores for immune cell types between different vaccine-immunized groups. (**B**) The proportion of different myeloid cell subclusters between different vaccine-immunized groups. (**C**) The proportion of different lymphoid cell subclusters between different vaccine-immunized groups.

Previous studies have demonstrated that the gut microbiota composition could shape the tumor immune microenvironment (TIME) by modulating specific immune cell infiltration [21]. We hypothesized that the tumor-resident microbiota might exhibit a similar function. We utilized xCell, a pioneering gene signature-based method, to characterize intratumoral immune cells and stromal cell infiltration from transcriptomic data. Each cell cluster (cancer-associated fibroblasts, epithelial cells including malignant and nonmalignant cells, immune cells including myeloid, natural killer, and T and B cells) was annotated based on the expression of curated gene signatures. A significantly higher number of CD8^+^ T cells, CD4^+^ T cells, memory B cells, Natural Killer T cells (NKT) and myeloid cells were found in the TIME of vaccine-immunized samples compared to controls (Figure 4A). These findings demonstrated that the abundance of the intratumoral microbiota is typically associated with an activated TIME, potentially enhancing the therapeutic efficiency of vaccine treatment. To better understand the heterogeneity of the tumor immune microenvironment between vaccine-immunized and control samples, we subsequently identified signature genes for each immune subcluster and successfully developed lymphoid and myeloid signatures (Figure 4B,C). The proportion of effector memory CD4^+^ T cells (CD4^+^ Tem), CD 8^+^ T cells, central memory CD8^+^ T cells (CD8^+^ Tcm), effector memory CD8^+^ T cells (CD8^+^ Tem), NKT and memory B cells significantly increased in the group receiving CCL11-E6E7 vaccine immunization treatment. Our study further demonstrated that the E6E7 and control groups exhibited increased infiltration of Th1 and Th2 cells (Figure 4C). Myeloid compartment analysis further indicated heightened infiltration of dendritic cell (DC) subsets—including immature DCs (iDCs), plasmacytoid DCs (pDCs), and conventional DCs (cDCs)—alongside significantly increased numbers of both M1 and M2 macrophages in vaccine-treated tumors (Figure 4B). The increase in these cells enhances antigen presentation, thereby boosting the vaccine’s anti-tumor effects. Collectively, bulk RNA-seq data revealed a comprehensive differential and tissue-specific tumor-resident immune cell landscape between the vaccine-immunized and control samples.

## 3. Discussion

Emerging studies identify intratumoral microbiota as key modulators of the tumor immune microenvironment (TIME), particularly through regulation of innate immune activation and therapy responsiveness [22]. In this study, our work systematically investigated the composition and diversity of intratumoral microbiota and its effect on therapeutic DNA vaccine response in tumor animal models by analyzing the previously obtained transcriptomic data from the vaccine immunization study.

Transcriptomic profiling revealed vaccine-induced remodeling of tumoral microbial ecosystems, with marked abundance shifts in Proteobacteria and Firmicutes versus controls without vaccine immunization. Emerging evidence indicates that intratumoral bacteria, including specific members of the Proteobacteria phylum, can exert positive immunomodulatory effects. Research on breast cancer demonstrated that *Listeria* secretes α-galactosylceramide (α-GalCer), a potent immunostimulant that activates invariant natural killer T (iNKT) cells, thereby bridging innate and adaptive immunity [21]. Furthermore, research has shown that *Salmonella* enhances antitumor immunity by expressing the immunomodulatory ligand LIGHT (TNFSF14), which directly activates both CD4^+^ and CD8^+^ T cells, including breast, colon, and lung cancer models [21]. Significant differences in both α-diversity and β-diversity were found between the vaccine-immunized group and the control group. Analysis of the classified taxa gene revealed that the intratumoral bacterial communities were dominated by members of the phyla *Proteobacteria*, *Firmicutes* and *Actinobacteria*, which also correspond to the predominant phyla in the human gut microbiome, indicating that the intratumoral microbiota may originate from the gut microbiome [23,24]. In this context, the relevant presence of taxa that can be found in the vaccine treatment group, such as *Sphingobium*, *Sphingopyxis*, *Escherichia*, *Delftia*, *Erythrobacteraceae* and *Corynebacteriaceae* at the family level, which was also detected in the human gut [25,26]. Therefore, samples in the vaccine treatment group exhibited a unique microbial signature within their tumors, characterized by a specific bacterial family associated with the treatment response. We note a methodological caveat: the original RNA-seq experiment [7] did not include library-extraction blank controls sequenced in parallel, so residual reagent-derived contamination cannot be formally excluded. Nevertheless, all tumor samples in this study were processed in the same experimental batch with the same extraction and sequencing pipeline, and any background contamination should affect vaccinated and control tumors equally. The group-specific, taxon-level LEfSe signals (Figure 3) and the group-separating beta-diversity ordination (Figure 2C) are therefore unlikely to be explained by batch contamination alone; they are, however, reported as putative bacterial signals and will require confirmation by 16S rRNA FISH or parallel blank-control sequencing.

Furthermore, several recent studies on comprehensive intratumoral microbiota analysis reported a close relationship between the microbiota and TIME [27]. Petra and colleagues found that vaccination might influence the composition of the intestinal microbiota and that the intestinal microbiota might influence vaccine responses [28]. Previous studies reported that tumor microbiome composition and diversity could affect immune cell infiltration, ultimately influencing patient survival [29,30]. Emerging evidence suggests that certain *Proteobacteria* within the tumor microenvironment exhibit immunostimulatory effects, potentially enhancing antitumor immune responses. Comparative 16S rRNA gene sequencing of intratumoral microbiota in pancreatic ductal adenocarcinoma (PDAC) patients demonstrated distinct microbial profiles between short-term survivors (STS) and long-term survivors (LTS). The tumor microbiome of LTS patients exhibited higher α-diversity, with a dominant microbial signature comprising *Proteobacteria* (e.g., *Pseudoxanthomonas*). This specific bacterial consortium was strongly predictive of prolonged survival through its capacity to promote immune cell infiltration, T-cell activation, and enhanced antitumor immune responses at the tumor site [17]. Studies have demonstrated that *Proteobacteria* enhances antitumor immunity in a natural killer (NK) cell- and CD8^+^ T cell-dependent manner by promoting IL-2 expression [31,32]. We performed RNA-seq on available tumor tissues to unravel the infiltration of intratumoral immune cells and crosstalk between the intratumoral microbiota and tissue-resident immune cells. The infiltration of CD8^+^ T cells, CD4^+^ T cells, memory B cells, Natural Killer T cells (NKT) and myeloid cells (DCs) was more abundant in the TIME of vaccine immunization samples compared to the control. Despite the close relationship between immune cell infiltration, vaccine treatment response, and intratumoral microbiota signatures, we did not detect a more specific or direct influence.

Further studies should focus more on exploring the mechanism by which the intratumoral microbiota may infect immune cells and the potential factors playing critical roles in the evaluation of the cross-reactivity between the immune cells recognizing tumor neoantigens and the tumor-resident microbiome. This may help understand the mechanisms by which bacteria exert immuno-activating effects and promote research into novel therapeutic strategies for cancer [33,34]. The underlying mechanisms need to be further evaluated.

In summary, our study elucidates the presence of an intratumoral microbiota associated with the vaccine treatment response in mice with cancer. The enrichment of the phyla *Proteobacteria* and a concomitant reduction in *Firmicutes* and *Actinobacteria* signatures result in a favorable response to cancer immunotherapy. The composition and diversity of the intratumoral microbiome could influence the infiltration of T cells and myeloid cells, thereby enhancing vaccine responses. This indicates potential clinical implications of intratumoral microbiota for cancer immunotherapy. Future investigations are warranted to elucidate the detailed mechanisms related to the tumor-resident microbiota and dynamic alterations.

## 4. Materials and Methods

### 4.1. Datasets

In this study, we analyzed RNA sequencing (RNA-seq) data from a prior investigation examining the effects of a DNA vaccine incorporating HPV16 E6/E7 antigens fused with the chemokine CCL11 on tumor immunity. Specifically, the dataset comprises transcriptomic profiles derived from the tumor tissues of C57BL/6N female mice. Tissues were harvested 14 days post-immunization (either with the CCL11-BEE7 vaccine or adjuvant control) and subsequent challenge with TC-1 tumor cells to gauge vaccine-induced immune activation and tumor growth suppression. Sample sizes per group are reported in [7].

### 4.2. Microbiome Data Acquisition

Our study employed PathSeq, a bioinformatics pipeline, to analyze microbial community data from sequencing reads. We improved the accuracy of downstream analyses by removing low-quality, low-complexity, host-derived, and duplicate reads using Apache Spark v4.2.0for efficient in-memory processing and the DUST algorithm for expedited filtering of low-complexity sequences. The remaining non-host reads were aligned to the reference microbial genomes and classified each read taxonomically using the BWA-MEM aligner. These pre-alignment filtering steps also remove a substantial fraction of likely reagent-derived, adapter-dimer and low-complexity contaminating sequences before taxonomic assignment, which materially reduces the background against which true microbial reads are called. Finally, a report is generated containing microbial abundance estimates at each taxonomic level.

### 4.3. Estimating Microbial Diversity

The alpha diversity within each sample was determined using the Shannon index and observed taxonomic features. This index simultaneously assesses both the richness and relative abundance of the microbial communities within each sample. The analysis of α-diversity, including richness estimators and diversity estimators, was performed using the Wilcoxon signed-rank test. The beta diversity analysis was carried out using principal coordinate analysis (PCoA) after calculating the distances between samples using the Bray-Curtis method, using the phyloseq R package (v. 1.22.3) with Bray–Curtis dissimilarities. We employed the LEfSe (LDA Effect Size) analysis to decipher the microbial biomarkers that significantly discriminated between groups. The LEfSe algorithm integrates linear discriminant analysis (LDA) with effect size measurements to identify features with the most pronounced differential abundance. Results were analyzed and plotted with the phyloseq R package (v. 1.30.0) and ggplot2 (v3.4.0). Differentially abundant taxa were considered significant if the *p*-value was less than or equal to 0.05.

### 4.4. Immune Deconvolution Methodology

Cell-type deconvolution is a computational method applied to bulk RNA-seq data to estimate the abundance of cell types in samples and is primarily used in the context of immune cells. In this study, we employ xCell 2.0 [35], a cutting-edge gene signature-based method, to deconvolute the complex cellular composition within tissues and infer the presence and proportions of 64 distinct immune and stromal cell types [22]. In this context, we utilize xCell to compare the immune cells between samples obtained from vaccinated samples and the control.

## 5. Conclusions

These findings establish that therapeutic HPV vaccination elicits specific, measurable alterations in intratumoral bacterial communities that parallel enhanced immune activation within the tumor microenvironment. The identification of vaccine-responsive microbial signatures—characterized by elevated Proteobacteria abundance and reduced Firmicutes and Actinobacteria representation—provides a mechanistic basis for microbiota-mediated modulation of antitumor immunity. Furthermore, the observed coordination between microbial community restructuring and dendritic cell maturation, together with effector lymphocyte recruitment, suggests that intratumoral bacteria may function as endogenous adjuvants to amplify vaccine-induced immune responses. These insights position intratumoral microbiota profiling as an actionable strategy for predicting immunotherapy outcomes and warrant future investigation into microbiota-targeted interventions to optimize therapeutic efficacy in HPV-associated malignancies.

## Data Availability

All the data relevant to the study are included in the previous article [7] and are available upon request from the corresponding author.

## Supplementary Materials

The following supporting information can be downloaded at https://www.mdpi.com/article/10.3390/ijms27219663/s1.
